# Progesterone and Its Metabolites Play a Beneficial Role in Affect Regulation in the Female Brain

**DOI:** 10.3390/ph16040520

**Published:** 2023-03-31

**Authors:** Małgorzata Stefaniak, Ewa Dmoch-Gajzlerska, Katarzyna Jankowska, Artur Rogowski, Anna Kajdy, Radosław B. Maksym

**Affiliations:** 1 Department of Obstetrics and Gynecology Didactics, Medical University of Warsaw, ul. Litewska 14/16, 00-575 Warsaw, Poland; 2 Department of Endocrinology, Centre of Postgraduate Medical Education, ul. Cegłowska 80, 01-809 Warsaw, Poland; 3 Department of Minimally Invasive and Endoscopic Gynecology, Military Institute of Medicine, ul. Zegrzyńska 8, 05-119 Legionowo, Poland; 4 1st Department of Obstetrics and Gynecology, Centre of Postgraduate Medical Education, ul. Żelazna 90, 02-004 Warszawa, Poland

**Keywords:** progesterone, allopregnanolone, progestins, mood, affect, luteal phase deficiency, pregnancy, premenstrual dysphoric disorder, premenstrual syndrome, maternal postpartum depression

## Abstract

Premenstrual dysphoric disorder is a female affective disorder that is defined by mood symptoms. The condition is linked to unstable progesterone concentrations. Progestin supplementation is given in cases of threatened or recurrent miscarriage and for luteal phase support. Progesterone is essential for implantation, immune tolerance, and modulation of uterine contractility. For a long time, the administration of progestins was associated with an unfavorable impact on mood, leading to negative affect, and, therefore, was contraindicated in existing mood disorders. Establishing the role of the natural progesterone derivative allopregnanolone in advances in the treatment of postpartum depression has shed new light on the general pathophysiology of mood disorders. Allopregnanolone directly interacts with gamma-aminobutyric acid type A (GABA-A) receptors even at nanomolar concentrations and induces significant anti-depressant, anti-stress, sedative, and anxiolytic effects. Postpartum depression is caused by a rapid drop in hormones and can be instantly reversed by the administration of allopregnanolone. Premenstrual dysphoric disorder can also be considered to result from insufficient neuroactive steroid action due to low progesterone derivative concentration, unstable hormone levels, or decreased receptor sensitivity. The decrease in progesterone levels in perimenopause is also associated with affective symptoms and an exacerbation of some psychosomatic syndromes. Bioidentical progesterone supplementation encounters several obstacles, including limited absorption, first-pass effect, and rapid metabolism. Hence, non-bioidentical progestins with better bioavailability were widely applied. The paradoxical, unfavorable effect of progestins on mood can be explained by the fact that progestins suppress ovulation and disturb the endocrine function of the ovary in the luteal phase. Moreover, their distinct chemical structure prevents their metabolism to neuroactive, mood-improving derivatives. A new understanding of progesterone-related mood disorders can translate the study results from case series and observational studies to cohort studies, clinical trials, and novel, effective treatment protocols being developed.

## 1. Introduction

Mental health issues are a serious social problem and a medical challenge worldwide. Women are more at risk of affective disorders [1]. Their susceptibility to these disorders changes with age, although mood disorders, age, and gender patterns have been observed, especially before menopause [2]. Epidemiological data shows that affective disorders occur about twice as often in women than in men, beginning in the reproductive period [3]. Copious scientific evidence points to ovarian hormone activity as a mediator of this difference [4]. The differences in female susceptibility to affective disorders are not static. Still, they may be stronger at certain times of the endocrine cycle and at certain stages of life when ovarian hormone levels are exceptionally high or variable, for instance, during adolescence. Research also indicates that the cyclical changes in the level of ovarian hormones cause periodic fluctuations in the mechanisms responsible for the connections between cells and neural networks [5]. Premenstrual dysphoric disorder (PMDD) is an example of a female affective disorder that is defined by mood symptoms and an increased vulnerability to stress, which may lead to severe disabilities.

Identifying the risk factors of depression symptoms helps women and their clinicians understand the potential course and plan of the therapeutic procedure. Mood and anxiety disorders that occur during the transitional periods in the reproductive period (including the menarche, menstrual cycle, pregnancy, and the postpartum period, as well as the perimenopause) may be subject to further observation in terms of the role of the sex steroids. Progesterone and its metabolites play an essential part in inducing mood disorders in women with menstrual disorders, and estradiol probably strengthens progesterone-invoked dysphoria [6]. Disturbances in the level of progesterone concentration may result from a luteal phase deficiency associated with a spectrum of ovulatory disorders, which are the result of a decreased ovarian reserve, non-ruptured follicle luteinization syndrome, or other metabolic or age-dependent disorders [7]. There is a membrane progesterone receptor in the brain cells, which is related to the control of emotions, among others, in the amygdala. It has been suggested that its malfunction may contribute to emotional dysregulation in women with PMDD [8,9]. Sex steroids also affect the renin-angiotensin-aldosterone system, which is most likely associated with certain somatic symptoms of premenstrual disorders, such as bloating and edema. Progesterone is quickly metabolized to pregnenolone and allopregnanolone, which act as positive allosteric modulators of the receptor for gamma-aminobutyric acid type A (GABA-A) [9,10]. At the same time, modulator activity increases the transmission of the primary neurotransmitter, which has a sedative effect on brain activity. Both pregnenolone and allopregnanolone have antidepressant and anxiolytic effects [9,10]. Data show that women with premenstrual disorders have a reduced plasma allopregnanolone content and a decreased reactivity of the GABA-A receptor to progesterone metabolites [9,10]. Due to the different chemical structures, artificially synthesized progestins will not undergo systemic and local metabolism to become active compounds. They are usually excreted in the urine in unchanged form, or their metabolites are inactive; therefore, they will not exert a similar action to natural progesterone. In theoretical considerations and clinical practice, the effects of bioidentical progesterone should be clearly distinguished from chemical compounds with different chemical structure and metabolism.

## 2. Results

### 2.1. The Role of Progesterone and Its Metabolites in Luteal Phase Deficiency

Recent research into progesterone provides important insights into the physiological role and clinical significance of this hormone [11]. Progesterone action is the key physiological element of the menstrual cycle. It also plays an important role in the development of the mammary gland and influences the function of the central nervous system and the cardiovascular system. It predominates in the luteal phase. Just after ovulation, progesterone secretion is stable and does not correlate with luteinizing hormone (LH) pulses, while in the middle and late luteal phases, progesterone secretion is episodic and correlates well with pulsatile LH release. During this period, the frequency and amplitude of LH pulses gradually decrease [12]. A decreased plasma progesterone concentration in the luteal phase may predict the occurrence of premenstrual syndrome (PMS); however, some clinical studies failed to provide clear evidence for progesterone being an effective treatment for premenstrual syndrome (PMS) [13,14]. It is worth noting that this meta-analysis only concerns two trials. Only one of them was rated as having a low risk of reporting bias because the study included all the outcomes of interest in the review. Additionally, the second study was not fully blinded and used relatively low (200–300 mg) and—as we know today—insufficient doses of progesterone. Moreover, it should also be noted that the exact time of ovulation was not determined, and progesterone was used on the basis of observations of previous menstrual cycles. It is good to remember that initially, the PMS treatment with progesterone that was advised in the 1940s recommended that the onset and dose of administration of progesterone should be tailored to each woman, increasing the number of 400 mg suppositories to as many as six per day during the luteal phase. Not only the high dose but also the route of administration, ensuring absorption, as well as a stable level of progesterone, are key to treatment effectiveness [15]. Nevertheless, in a high-dose study included in the meta-analysis, a statistically significantly greater improvement was recorded in the supplemented group in the per-protocol analysis [13].

On the other hand, in a recent study on 20 women with PMS and 21 healthy women, researchers concluded that when symptoms are redefined as perimenstrual rather than premenstrual, there is an association with both lower steady-state progesterone levels and a luteal phase deficiency [14].

In a meta-analysis of the Cochrane consortium on progesterone supplementation, it was found that progesterone reduces the incidence of miscarriages in women with unexplained recurrent miscarriages [16]. Although, in the past, the problem of treating a miscarriage possibly caused by the use of progesterone was the subject of numerous controversies and significant geographical differences in medical practice around the world. A recent meta-analysis has shown that, at least for patients with a history of miscarriage who bled in their next pregnancy, the administration of a high dose of vaginal progesterone (2 × 400 mg) is undoubtedly beneficial. Not only the high dose but also the route of administration, ensuring absorption without the first-pass effect, as well as its stable levels, are important for its effectiveness. This has already led to a change in the national medical recommendations in the United Kingdom (UK) [17]. It is not optimistic that, until convincing scientific evidence was obtained, the standard treatment in this indication was suspended for many years, which resulted in an additional loss of over 8000 pregnancies per year in the UK alone.

Unstable progesterone levels in premenstrual dysphoric disorders are most commonly explained by a luteal phase deficiency (LPD). LPD can be found in diverse ovulatory disorders originating from a diminished ovarian reserve and a luteinized unruptured follicle to a spectrum of polycystic ovary syndrome [7]. LPD can also frequently occur during controlled ovarian stimulation (COS) protocols and controlled ovarian hyperstimulation (COH) programs. Luteal phase deficiency was first described as a possible cause of infertility in 1949 by Georgiana Seegar Jones [18]. The pathophysiology of LPD may involve several different mechanisms that ultimately affect the development and function of the endometrium. LPD has been described as a condition in which the production of the ovarian hormone is not high enough or long enough to maintain a functional secretory endometrium and allow the proper implantation and growth of the embryo. A short luteal phase is associated with low levels of follicle-stimulating hormone (FSH), a low estradiol level in the follicular phase, an altered FSH/LH ratio in the follicular phase, and an abnormal pulsation of FSH and LH [19]. These follicular phase abnormalities are associated with the subsequent reduction in luteal estrogen and progesterone secretion [20,21,22,23]. Alternatively, LPD may develop on the receptor level, as a result of an abnormal endometrial response to an adequate hormone concentration [24,25]. Idiopathic LPD denotes a luteal phase abnormality in the absence of an identifiable disease process. Difficulties in diagnosing LPD result from inconsistent and unreliable diagnostic criteria, as well as a low availability of appropriate diagnostic methods. The diagnosis of LPD is usually clinically made by an assessment of the length of the luteal phase. Many diagnostic tests have been proposed, including clinical, biochemical, and histological tests, but none of them have been able to reliably differentiate fertile and infertile women [26,27,28,29]. In order of increasing invasiveness, the methods used to diagnose LPD include the diagnosis of a shortened luteal phase based on the length of the menstrual cycle, the basal body temperature (BBT) graph, or urine LH surge kits, the measurement of progesterone derivatives in urine, the measurement of single or multiple serum progesterone levels, as well as an endometrial biopsy [7].

In the treatment of LPD, it is important to recognize any underlying disorders and take a restorative approach. While, in the absence of dysfunction, therapeutic management becomes empirical, in the past, the goals of empirical treatment were to stimulate ovulation, increase endometrial receptivity, and support implantation and early pregnancy development. Empirical strategies included progesterone supplementation, progesterone plus estradiol supplementation, luteal human chorionic gonadotropin (hCG), and various ovarian stimulation regimens [7].

A wide variety of progesterone and progestogen functions that were applied in clinical practice have already been discovered and described [7,8]. However, the role of progesterone in maintaining the luteal phase remains controversial due to its wide and empirical cross-sectional clinical use, ranging from natural ovulatory cycles to assisted reproductive techniques (ARTs). However, no evidence has been found that progesterone is beneficial in the treatment of LPD. No randomized, case-control studies have been found investigating progesterone supplementation in women with LPD. The research conducted so far has only concerned progesterone supplementation in cases of repeated miscarriages, which could theoretically overlap with LPD due to insufficient progesterone support in early pregnancy [7]. In clinical practice, progesterone supplementation is the suggested treatment for LPD. Despite being frequently used, there is no published evidence that it improves pregnancy outcomes in natural cycles [7]. Discussions on the protocols for progesterone administration, especially the route of administration, dose, and timing of administration, and the potential relationship with other drugs, remain open and further research is needed. It has been shown that an advanced reproductive age is associated with a luteal phase deficiency (LPD). Studies have confirmed a reduced production of progesterone and disorders of progesterone, as well as estradiol metabolites, in the luteal phase in women of late reproductive age [25,26,30,31].

### 2.2. The Role of Progesterone and Its Derivatives in the Regulation of Emotional Disorders in Women

Progesterone supplementation is conventionally administered for threatened or recurrent miscarriages and for maintaining the luteal phase, especially during assisted reproductive cycles. Progesterone is necessary for implantation, immune tolerance of the embryo, and modulation of uterine contractility; therefore, its action is essential in early pregnancy. While high-dose progesterone was used years ago in the treatment of PMS—and the only properly conducted studies show some improvement in effect with progesterone—it is commonly believed that there is insufficient evidence for its use in this indication [13]. The long-term administration of non-bioidentical progestins has been related to adverse effects on mood, and they have been contraindicated in existing mood disorders. The temporal relationship between PMDD symptoms and the variation in luteal progesterone levels, as well as evidence from ovarian suppression studies, make this hormone and its metabolites important for the symptomatology of PMDD. Fluctuations in progesterone levels appear to be key to the development of the disorder. Indeed, the suppression of ovulation leads to a reduction in progesterone levels, and what could be more important is its fluctuation, leading to a remission of symptoms. On the other hand, a single oral or vaginal daily dose (every 24 h) of progesterone induces fluctuation and causes symptoms to reappear [8], as a rapid peak in concentration is achieved after a single administration, and levels can drop almost to zero for part of the day. Pharmacodynamic analyses of oral and vaginal formulations suggest that to achieve stable levels, the dose must be repeated several times a day, depending on the route of administration. Progesterone derivatives, such as pregnenolone and allopregnanolone, act in the regions of the brain that are responsible for processing emotions as allosteric modulators of the GABA-A receptor [9,10]. Establishing the role of the progesterone derivative allopregnanolone in the development of postpartum depression treatment has shed new light on the general pathophysiology of mood disorders. Allopregnanolone directly interacts with GABA-A receptors, even at nanomolar concentrations, and has significant anti-depressant, anti-stress, sedative, and anxiolytic effects [8,9]. Allopregnanolone and other progesterone metabolites act as positive modulators on GABA-A receptors. Enzyme 20α-hydroxysteroid dehydrogenase catalyzes the conversion of GABA-active progesterone metabolites into inactive metabolites. Protracted downregulation of the δ subunit-containing GABA-A receptor during the postpartum period has been associated with peripartum depression-like behavior in preclinical models. Altogether, as postpartum blues is a risk factor for PPD, this suggests that a maladaptive homeostatic plasticity of GABAergic sensitivity to neurosteroids during pregnancy and postpartum are key contributors to PPD [32,33]. Allopregnanolone (ALLO), synthesized by glutamatergic neurons of the olfactory bulb, frontal cortex, hippocampus, and amygdala, modulates GABA action at synaptic or extrasynaptic GABA-A receptors. These receptors are located on the dendritic shafts or cell bodies of the above-mentioned glutamatergic neurons by an autocrine mechanism or, more likely, by this neurosteroid reaching GABA-A receptor intracellular sites through lateral membrane diffusion [32].

The opposite relationship between depressive symptoms and progesterone exposure supports the recent discovery of the antidepressant effect of allopregnanolone in the treatment of postpartum depression. The high effectiveness of the drug has led to its approval by the Food and Drug Administration (FDA) as a medication for postpartum depression [34].

Two double-blind, randomized, placebo-controlled, phase III trials were conducted at 30 clinical research centers and specialized psychiatric units in the USA. Eligible women were aged 18–45 years, 6 months postpartum or less at screening, with postpartum depression and a qualifying 17-item Hamilton Rating Scale for Depression (HAM-D) score (≥26 for study 1; 20–25 for study 2). Women with renal failure requiring dialysis, anemia, known allergies to allopregnanolone or progesterone, or a medical history of schizophrenia, bipolar disorder, or schizoaffective disorder were excluded. Patients were randomly assigned (1:1:1) to receive a single intravenous injection of either brexanolone 90 μg/kg per h (BRX90), brexanolone 60 μg/kg per h (BRX60), or a matching placebo for 60 h in study 1, or (1:1) BRX90 or a matching placebo for 60 h in study 2. The patients, study team, site staff, and principal investigator were masked to treatment allocation. The primary efficacy endpoint was the change from baseline in the 17-item HAM-D total score at 60 h, assessed in all patients who started the infusion of the study drug or placebo, had a valid HAM-D baseline assessment, and had at least one post-baseline HAM-D assessment. The safety population included all randomized patients who started the infusion of the study drug or a placebo. The patients were followed up until day 30. The administration of a brexanolone injection for postpartum depression resulted in significant and clinically meaningful reductions in the HAM-D total score at 60 h compared with the placebo, with a rapid onset of action and a durable treatment response during the study period [34].

As a drug for other mood disorders related to reproductive hormones, the effectiveness of allopregnanolone in the treatment of postpartum depression challenges the previous assumptions that progestins always exert a negative effect on mood. Allopregnanolone may act as a neurosteroid, mediating the protective effect of peripheral progesterone on mood by the direct modulation of gamma-aminobutyric acid (GABA) receptors.

However, the relationship between ovarian progesterone production, centrally active allopregnanolone, and GABA regulation still remains to be established in women with hormonally sensitive mood disorders. Research results indicate a beneficial effect of progesterone and its metabolites on mood in women with hormonally linked mood disorders [34]. Furthermore, as in the case of GABA-A, progesterone receptors are present throughout the brain in sites where they influence the neural circuits that are crucial for cognitive and affective processing [35].

Progesterone receptors (PR) are broadly expressed throughout the brain, with no apparent restriction to specific cell types. Nevertheless, PR expression may vary depending on the brain region, cell type, or hormonal status. Both of the classical PR isoforms (PR-A and PR-B) are expressed in the hippocampus and frontal cortex [36].

The GABAergic system also participates in major depression [37]. The GABA-A receptor mediates the majority of rapid (1–100 ms) synaptic inhibition in the mammalian brain, and allopregnanolone (ALLO) and pregnenolone (PREG) exert both anxiolytic and anesthetic effects by enhancing GABA-stimulated chloride conductance. This enhanced conductance serves to hyperpolarize postsynaptic membranes and results in neuronal inhibition. Recent evidence suggests that specific neurosteroids ‘fine-tune’ neural inhibition via the GABAergic system [38]. In another recent study, the ability of progesterone to influence cognition and memory of biologically salient stimuli was investigated in healthy young women [39]. Here, a single dose of progesterone was orally administered to women who were then asked to memorize and recognize faces while undergoing functional magnetic resonance imaging. The results revealed that progesterone decreases recognition accuracy without affecting reaction times. Progesterone also decreased amygdala and fusiform gyrus activity elicited by faces during memory encoding, supporting the conclusion that progesterone alters memory function by influencing amygdala activity [38,39].

Postpartum depression is caused by a sudden decrease in steroid hormones after the expulsion of the placenta and can be immediately reversed by administering allopregnanolone [9]. The premenstrual dysphoric disorder can also be considered to result from insufficient neuroactive steroid action due to fluctuating progesterone derivative concentration or decreased receptor sensitivity. This is why oral contraceptive pills (OCPs) are considered the first-line drug for the treatment of PMDD [40]. There is mixed evidence of their efficacy in the treatment of PMDD [41]. Hormone monotherapy appears to be less effective than combination therapy. As mentioned above, a meta-analysis of the Cochrane review of progesterone use in PMS found no strong evidence for the effectiveness of progesterone alone, mainly due to the lack of reliable research on this topic [13].

In turn, a study conducted among 122 healthy women of reproductive age showed that decreased levels of aggression, irritability, and fatigue were observed with increased levels of progesterone in the luteal phase, and, in addition, the peak level of progesterone in the luteal phase negatively correlated with the same symptoms [42]. However, in many studies, there were no significant differences in the levels of estradiol and progesterone between women with PMS and those without [43,44,45]. A specific profile of luteal phase progesterone is associated with the development of premenstrual symptoms [43]. In other research, patients with PMS had lowered [46,47] or increased [48,49] progesterone levels. Gailliot et al. hypothesized that premenstrual mood swings are the result of impaired self-control due to insufficient energy resources in the luteal phase when energy requirements are increased due to intense metabolic changes in the genitourinary system [50]. It has been found that insufficient energy resources reduce the level of progesterone and, thus, lower the level of neuroactive metabolites of progesterone, such as allopregnanolone. The levels of these metabolites have been found to influence mood and behavior in women [44]. Studies have shown a positive correlation with progesterone levels during the menstrual cycle and that these levels are lower in PMS patients in a case-control study [44,45]; thus, a model of the biphasic effect of progesterone metabolites on mood was obtained. Accordingly, it was found that a low concentration of allopregnanolone through the GABA-A system intensifies negative mood changes, such as irritability and aggression, while a high concentration has a calming effect on the mood. However, direct evidence for this model in women of reproductive age is sparse. These observations confirm the results of a study that found that negative mood symptoms in women with PMDD are caused by the paradoxical action of allopregnanolone mediated by the GABA-A receptor [9].

GABA-A receptor modulators are known to induce adverse, anti-anxiety effects at low concentrations, whereas at higher concentrations, they show beneficial sedative properties. The mechanism causing the paradoxical response may be similar in women responding to positive GABA-A receptor modulators and in women with PMDD. The severity of these mood symptoms in women is related to serum allopregnanolone concentrations on an inverted U-shaped curve. Negative mood symptoms occur when the serum allopregnanolone concentrations are close to the endogenous luteal phase levels, whereas low and high concentrations have less of an effect on mood. Low to moderate concentrations of progesterone/allopregnanolone in women increase amygdala activity (as measured by functional magnetic resonance imaging (fMRI)) similarly to the changes observed during anxiety reactions. Higher concentrations result in reduced amygdala activity, as observed during treatment with benzodiazepines with sedative anti-anxiety effects. This agrees with animal studies, which show an association between the changes in the alpha4 and delta subunits of the GABA-A receptor and the anxiety-like effects of allopregnanolone [9].

Furthermore, evidence suggests that PMS-like symptoms may appear with cyclical and continuous progesterone treatment [51]. The Roomruangwong et al. research has shown that a reduced concentration of sex hormones, mainly progesterone and, to a lesser extent, estradiol, averaged over the menstrual cycle, allows the prediction of the presence of PMS and its severity [14]. Earlier studies investigating the relationship between progesterone levels and the development of PMS have produced controversial results. The discrepancies in some of the studies mentioned above may be due to the fact that the worsening of the symptom may be preceded by a change in steady-state progesterone levels combined with changes in progesterone levels over time in the diffuse delay model (with current and delayed sex hormone values).

When it comes to monophasic (same hormone dose per day) versus multiphase (varying hormone levels over a 21- or 28-day cycle) oral combined pills (OCP), monophasic pills are generally recommended to avoid hormone fluctuations that can contribute to worsening mood in PMDD [52,53]. However, few studies have compared monophasic and multiphasic OCP. There is also evidence that progestins in other hormonal contraceptives, such as patches, vaginal rings, progestagen implants or injections, or hormone-containing intrauterine devices (IUDs), may worsen PMDD symptoms [54]. In case of their ineffectiveness, as well as in depression, selective serotonin reuptake inhibitors (SSRI) are used, the action of which is based on the inhibition of the reabsorption of serotonin by neurons [52]. Meta-analyses have shown that SSRIs are effective in treating PMDD, reducing the symptoms significantly more than a placebo [46,47], including both mood and physical symptoms [55,56,57]. SSRI may be beneficial in psychiatric disorders because, in doses that are inactive on serotonin (5-HT) reuptake mechanisms, they increase the bioavailability of neuroactive GABAergic steroids. The Pinna et al. studies provide evidence suggesting that fluoxetine upregulates endogenous brain stores of allopregnanolone and regulates GABAergic tone and related behaviors by a mechanism that may be independent of the modifications of 5-HT reuptake mechanisms [58].

A decrease in progesterone levels in the perimenopause is also associated with affective symptoms and an exacerbation of some psychosomatic syndromes, such as irritable bowel disease. Bioidentical progesterone supplementation encounters several obstacles, including limited absorption, first pass effect, and rapid metabolism. Therefore, non-bioidentical progestins with better bioavailability are widely applied. The paradoxical, unfavorable effect of progestins on mood can be explained by the fact that progestins suppress ovulation and disturb the endocrine function of the ovary in the luteal phase and lead to a remarkable decrease in progesterone secretion. Moreover, the lack of bioidentity prevents their metabolism to neuroactive mood-improving derivatives. The development of more efficient delivery systems for bioidentical progesterone or causative therapies that could improve internal progesterone secretion is desirable. A novel understanding of progesterone-related mood disorders can lead to the translation of the study result into clinical practice [59,60,61].

In 2017, an open proof-of-concept study was conducted using a 60-h allopregnanolone infusion. The drug was infused for PPD, which showed that the mean depression scores were reduced to a level corresponding to symptom remission [62]. Following the publication of these results, further studies have emerged from two double-blind, randomized, placebo-controlled phase III studies, again showing a positive effect in PPD [34].

The Kaltsouni et al. research results on the role of progesterone in the symptomatology of PMDD suggest a beneficial effect of the antagonism of progesterone receptors and, consequently, anovulation, and this may significantly affect the regulation of emotions, that is, greater antero-temporal activity in response to provocative stimuli [63]. Bixo et al. showed promising results for the GABA-A receptor modulating antagonist as a potential drug in the treatment of PMDD. This exploratory randomized trial assessed the efficacy and safety of sepranolone (UC1010) in the treatment of PPD in one phase II clinical trial (PPD-202A) and two phase III clinical trials (PPD-202B and PPD-202C) [64]. The participants were 26 healthy women in a pharmacokinetic phase I study, and 126 women with PMDD in a phase II study. The diagnosis followed the criteria for PMDD in the DSM-5 using the Daily Record of Severity of Problems (DRSP) and Endicott’s algorithm. Inclusion criteria for both study parts were women of 18–45 years of age, essentially healthy, with regular menstrual cycles, and using non-hormonal contraception. The subjects were randomized to treatment with UC1010 (10 or 16 mg) subcutaneously every second day during the luteal phase or a placebo during one menstrual cycle. The results showed that UC1010 reduced PMDD symptoms significantly better than a placebo, based on per-protocol analysis. The potential effect of UC1010 treatment is promising if it is administered in a more optimal manner. UC1010 was well tolerated, with no clinically significant changes in the safety variables [64].

Similar analyses were performed by Bäckström et al. and showed a decreasing effect of sepranolone on symptoms, weakness, and anxiety in women with PMDD, especially at the 10 mg dose. Sepranolone was well tolerated, and no safety concerns were identified [65]. The participants were 206 women with PMDD from 12 European centers and were randomized in a parallel double-blind study and treated with a placebo and sepranolone 10 mg and 16 mg. The patients were administered sepranolone subcutaneously every 48 h during the 14 premenstrual days of three consecutive menstrual cycles. After obtaining informed consent, the PMDD diagnosis was confirmed according to the DSM-5 and verified with two menstrual cycles of daily symptom ratings using the Daily Record of Severity of Problems (DRSP) scale in an eDiary. The inclusion and exclusion criteria stipulated that the women should be essentially healthy, not pregnant, have no ongoing psychiatric disorder, or be taking interfering medications, and have regular menstrual cycles. The study’s primary endpoint was the total symptom score (Sum21, the score for all 21 symptom questions in the DRSP). In the prespecified statistical analysis, the average score of the 5 worst premenstrual days in treatment cycles 2 and 3 was subtracted from the corresponding average score in the two diagnostic cycles. The treatment effects were tested using analysis of variance in a hierarchal order, starting with the combined active sepranolone treatments vs. the placebo [65].

**Figure 1:**
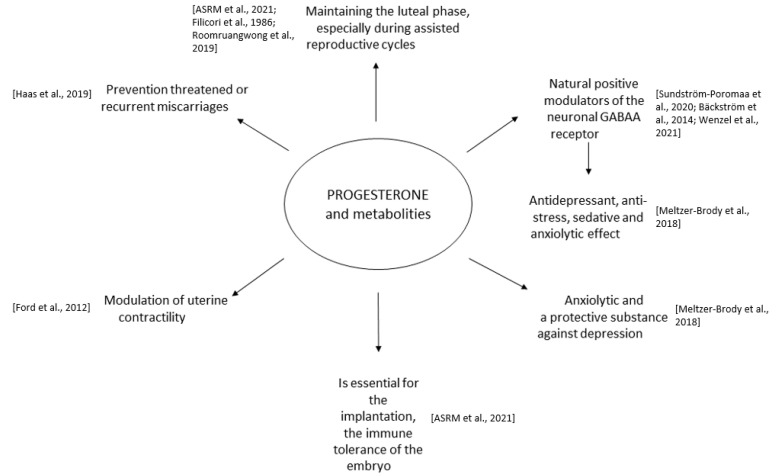
Pharmacodynamic profile of progesterone [7,8,9,10,12,13,14,16,34].

**Figure 2:**
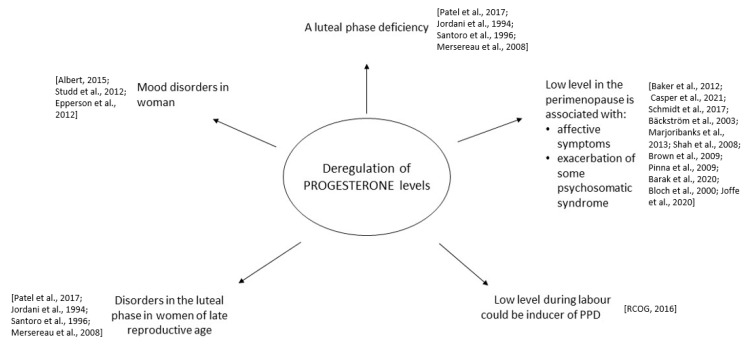
Deregulation of progesterone levels and connection with health disorders [25,26,30,31,40,51,52,53,54,55,56,57,58,59,60,61,67,68,69].

The new mechanism of action of allopregnanolone agonists on GABA-A receptors is a promising innovation in the treatment of depression disorders. Their advantage is a quick onset of remission compared to standard therapies, which may be a niche as inducing drugs or a bridge to SSRI maintenance therapy. Given the role of endogenous allopregnanolone in PPD, the benefits of these drugs may outweigh the costs and serious risks of untreated PPD. On the other hand, in PMDD, the mechanism of action of allopregnanolone agonists reflects the mechanism of action of benzodiazepines, which are no longer recommended and could potentially indicate no benefit in this indication. These data are encouraging, but more research is needed on the topic of combination drugs [66]. The pharmacodynamic profile of progesterone and metabolities, and the connection to the pathologic deregulation of progesterone levels are presented in Figure 1 and Figure 2.

### 2.3. Role of Progesterone and Allopregnanolone in Affective Disorders in The Perinatal Period

Mood disorders affect women unproportionally in their lifetime. Women are exposed to depression in adolescence as well as in the premenstrual, perinatal, and perimenopausal periods [67]. This especially applies to pregnant and postpartum women. In the first trimester of pregnancy, there are rapid changes in the endocrine system (an increase in the number of estrogen and progesterone receptors) [68]. Estradiol and progesterone affect the system of neurotransmitters (serotonin, dopamine, and norepinephrine), causing emotional disturbances [69]. Both estrogen and progesterone exert acute effects on synaptic physiology through the activation of multiple intracellular signaling pathways, including the MAPK/ERK and the Akt pathway, which are both part of a non-genomic signaling cascade linked to the promotion of cell survival [69]. Sex hormones act on multiple levels simultaneously, as well as the interacting neurotransmitter systems that are largely interwoven. Sex hormones can impact dopaminergic neurotransmission via a multitude of mechanisms. The stimulating as well as inhibiting effects of estrogen on dopaminergic neurotransmission have also been documented. These conflicting findings are not surprising when considering the many aspects that can influence the impact of estrogen on the dopaminergic (DA) system, such as the dose and time of testing, the mode of administration, the duration of the exposure, and the time after the exposure.

Estrogen may produce its mentioned effects on cognition and mood, especially through the modulation of serotonergic function [69]. Estrogen can increase serotonin levels and decrease 5-HT reuptake [70], which allows 5-HT to remain in the synaptic cleft for longer and exhibit prolonged effects on the postsynaptic receptors.

In addition, women could be afraid of miscarriage or the social changes that are related to pregnancy [71]. Emotions usually stabilize during the second trimester. The last trimester of pregnancy is marked by a renewed increase in anxiety and uncertainty about the upcoming delivery. Due to the changes in external appearance, women’s physical self-esteem also lowers, which also affects the development of depression [72]. The commonness of affective disorders and the significant implications for women affect their quality of life and greatly reduce their ability to provide adequate care to the newborn [73]. Postpartum mood swings in women can occur as a result of excess stress, anxiety, and uncertainty. There are three main stages of mental disorders in the puerperium: baby blues, postpartum depression, and postpartum psychosis. In the twentieth century, Channi Kumar discovered that mental disorders are about 100 times more common in perinatal women than over the rest of their lives [74]. The emergence of this thesis has led to the initiation of careful observation of patients in terms of the occurrence of emotional disorders. The current knowledge about perinatal mental disorders is much greater than a dozen or so years ago, thanks to which the diagnosis of abnormalities allows for the introduction of appropriate therapy as early as possible, which in most cases is characterized by the desired effectiveness. Factors that may have a direct impact on the occurrence of postpartum mental disorders have been mentioned in the literature. They are, among others, biological indicators, which include hormonal changes such as changes in the level of progesterone, estrogens, prolactin, thyroxine, and cortisol. The concentration of these substances increases during pregnancy and decreases sharply after childbirth. The correlation between the levels of progesterone and estrogens with the increase in prolactin is very important during the first days of the puerperium because it determines the mood of a woman. The development of postpartum mental disorders is also greatly influenced by thyroxine (T4), the level of which is almost 50% lower after childbirth than in the third trimester of pregnancy. As a result, hypothyroidism occurs, and psychotic symptoms may then appear up to nine months after childbirth and are termed late-onset postpartum depression [74].

Among the biochemical factors causing postpartum depression is a decrease in the concentration of serotonin, tryptophan, and endorphins. Postpartum depression can be caused by a decrease in cyclic adenosine-3′,5′-monophosphate (cAMP)—a nucleotide made of ribose, adenine, and phosphate. Moreover, cAMP is an important intracellular second messenger in many physiological processes. A link has also been found between high calcium levels and the development of postpartum psychosis [75]. What is more, the results of the conducted research show that PMS is also an important predictor of peripartum depression [14,68,76,77].

However, research on the effects of progesterone in women suffering from PPD is sparse and inconclusive. Barak et al. hypothesized that the repeated administration of oral progesterone may increase the concentration of allopregnanolone in the central nervous system, which should alleviate the symptoms of PPD and constitute an alternative to brexanolone [59]. Bloch et al. simulated a postpartum hormonal state by inducing hypogonadism in euthymic women: eight with and eight with no history of PPD. Within 8 weeks, they were able to show that the progesterone drop was associated with an increase in depression in women with a history of PPD [60].

The influence of estradiol and progesterone variability on mood in perimenopausal women with depressive symptoms was investigated by Joffe et al. This study showed that mood instability in the perimenopausal period is caused by hormonal dysregulation in the menopausal period, which includes both estradiol and progesterone changes [61]. The findings of this study are consistent with the recent discovery of the antidepressant effect of allopregnanolone in the treatment of postpartum depression [34].

Depression during pregnancy can also be a cause of mental disorders in early childhood, problems with learning, and social issues [78]. Moreover, according to World Health Organization (WHO) data, depression during pregnancy is a strong risk factor for the development of postpartum depression [79], which can apply to 10–15% of women in a period of up to 12 months postpartum. In addition, a lack of proper treatment of depression in the expectant mother may have negative effects on the fetus (e.g., premature delivery, reduced birth weight, lower Apgar scores, and increased levels of stress hormones in the baby). An early and correct diagnosis may minimize the negative impact of depression on the health of both mother and child [80,81]. Considering the above, it seems particularly important to look for solutions that can safely minimize the risk of the development of depression.

The issue of depression in pregnancy and the puerperium is the subject of ongoing research. As with PMDD, attempts have been made to find differences in the levels of major sex steroids, estrogen, and progesterone. Progesterone has long been considered an anxiolytic and a protective substance against depression [82,83], and some studies suggest that progesterone withdrawal may be associated with PPD [84]. The current data on the role of progesterone and its metabolites in perinatal mood and anxiety disorders are inconclusive. This is most likely due to improper protocols for determining hormone levels and the researchers’ focus on the hormones themselves and not on the mediators, such as their metabolites [85].

Buckwalter et al. showed more mood disorders during an advanced pregnancy than in the puerperium; however, no connection with the progesterone level was presented in the research [86]. Other studies have shown that the drop in progesterone levels during labor was an inducer of PPD [60,84,87,88].

In the Heidrich et al. research, higher levels of progesterone have been found in women with symptoms of postpartum blues [89]. Inconclusive research indicates that there is no hard evidence that women who develop antenatal or postnatal depression have different levels of progesterone after delivery.

The relationship between progesterone and its metabolites and PPD has been directly investigated in a number of studies. The evidence for changes in allopregnanolone levels in mood disorders in pregnant and postpartum women is also inconclusive. Hellgren et al. found that women in late pregnancy with increased symptoms of depression had significantly lower levels of allopregnanolone than those with normal results [90], while Deligiannidis et al. found no relationship between the level of the hormone in late pregnancy or early postpartum and the development of PPD in a small sample [91]. The Hellgren studies have also found that a low allopregnanolone level correlates with a higher depression score in the third trimester of pregnancy and with greater disturbances in the emotional state of women in the second trimester of pregnancy [90]. Osborne et al., in their exploratory study, hypothesized that women who develop PPD will have lower allopregnanolone levels when measured in the second and third trimesters of pregnancy and confirmed a significant relationship between low allopregnanolone levels in the second trimester of pregnancy and the development of PPD in women with a history of mood disorders. However, the study had some limitations, including the size of the sample and the presence of confounding factors that may affect the obtained results [85]. Some evidence suggests changes in GABA-A receptors as a mediator of the relationship between allopregnanolone and mood in the perinatal period [92]. Maguire et al. found that increasing levels of allopregnanolone in pregnancy may reduce the regulation of GABA-A receptors, and the lack of adequate receptor regulation in pregnancy may be a risk factor for the development of PPD [92]. Research results in this area are still inconclusive, as they indicate contradictory effects—both increased and decreased levels—of allopregnanolone in the treatment of depression [90,93,94,95,96,97,98,99].

## 3. Future Perspectives

The ongoing discussions on the protocols for progesterone administration (route of administration, dose, and timing of administration relative to the time of ovulation, and the potential relationship with other drugs) indicate the need for further research in the area. It is still unknown if PMS, its severity, and its significant symptomatic factors are related to the steady-state levels coupled with the changes in sex hormones during the menstrual cycle. The dysregulation of progesterone and its metabolites seems to play an important role in mood disorders in women, although the nature of this role is still the subject of much debate. The studies cited above have shown that allopregnanolone plays a role in the affective symptoms related to the reproductive system; however, more research is needed to understand which part of this role is due to the differences in its levels (both absolute and fluctuations) and which part is due to receptor plasticity and from the interaction with other systems, such as the hypothalamic-pituitary-adrenal (HPA) axis and the immune system. So far, the results of meta-analyses concerning the lack of efficacy of progesterone in the treatment of PMS seem to be based on a rather weak basis. New light has been shed on the role of progesterone and its metabolite, allopregnanolone, in the regulation of affect in women thanks to groundbreaking work in the treatment of postpartum depression.

The role of other progesterone metabolites in mood disorders and anxiety symptoms is currently unknown. The discussed results may indicate future research directions that will allow a better understanding of the biological mechanisms underlying affective disorders in women.

## 4. Materials and Methods

This paper aims to provide clinicians with comprehensive data based on the most relevant and recent scientific research, which summarizes the current knowledge on progesterone and its metabolite, allopregnanolone, in the context of their role in affective disorders.

We conducted an investigation which was limited to the English language in order to obtain the required scientific papers. Articles published up to the end of 2021 were gathered, read, and carefully analyzed. A search was conducted on the Medline repository (via PubMed) using a combination of the following subjects or keywords: “luteal phase deficiency”, “premenstrual dysphoric disorder”, “progesterone”, “affective disorder”, “allopregnanolone”, and “postpartum depression”.

We carried out additional literature searches based on literature from selected studies. Preference was given to meta-analyses, systemic reviews, and randomized clinical trials (RCTs). In the absence of meta-analytical or RCT data, the final analysis included prospective, non-randomized studies followed by cohort studies and guidelines of medical societies. The characteristics and outcomes of the most important articles included in the review are provided in Table 1.

## 5. Conclusions

Traditionally, progesterone and progestins are believed to have a negative effect on women’s affect regulation. The knowledge so far has not been based on reliable scientific evidence, appropriate timing, or doses used for treatment. New research on this topic shows the diverse actions of progesterone and progestins. Of particular importance is the fact that bioidentical progesterone also acts through its metabolites, and progestins are often not metabolized into active molecules. A new understanding of these issues prompts new research and changes in clinical procedures.

## Figures and Tables

**Table 1:** Characteristics of the most important included articles.

Author	Publication Year	Population Size	Type of Study	Hormone Used	Objectives	Primary Outcomes	Statistical Significance
Ford et. al. [13]	2012	280	Systematic review	Progesterone	The objectives were to determine if progesterone has been found to be an effective treatment for all or some premenstrual symptoms and if adverse events associated with this treatment have been reported.	The trials did not show that progesterone is an effective treatment for PMS or that it is not.	No statistically significant difference
Roomruangwong et al. [14]	2019	41	Observational study	Quantitative determination of oestradiol and progesterone.	To examine associations between levels of progesterone and oestradiol during the menstrual cycle and PMS, considering different diagnostic criteria for PMS.	Lowered steady-state levels of progesterone, when averaged over the menstrual cycle, together with declining progesterone levels during the luteal phase, predict the severity of peri-menstrual symptoms.	>0.8
Haas et al. [16]	2018	2556	Systematic review	Progestogen	To assess the efficacy and safety of progestogens as a preventative therapy against recurrent miscarriage.	The meta-analysis of all women suggests that there is probably a reduction in the number of miscarriages for women given progestogen supplementation compared to placebo/controls (average risk ratio (RR) 0.69, 95% confidence interval (CI) 0.51 to 0.92, 11 trials, 2359 women, moderate-quality evidence).	Average risk ratio (RR) 0.69, 95% confidence interval (CI) 0.51 to 0.92, 11 trials, 2359 women, moderate-quality evidence.
Meltzer-Brody et al. [34]	2018	375	Double-blind, randomised, placebo-controlled, phase III trials	Breksanolon patients were randomly assigned (1:1:1) to receive a single intravenous injection of either brexanolone 90 μg/kg per h (BRX90), brexanolone 60 μg/kg per h (BRX60), or matching placebo for 60 h.	Assessed brexanolone injection (formerly SAGE-547 injection), a positive allosteric modulator of γ-aminobutyric-acid type A (GABAA) receptors, for the treatment of moderate to severe postpartum depression.	Administration of brexanolone injection for postpartum depression resulted in significant and clinically meaningful reductions in the HAM-D total score at 60 h compared with placebo, with a rapid onset of action and durable treatment response during the study period.	Statistically significant
Ziomkiewicz et al. [42]	2012	122	Observational study	Saliva samples were assayed for progesterone concentrations.	Assayed for progesterone concentrations and mood intensity scores were used to calculate behavioral indices.	Women with low aggression/irritability and fatigue had consistently higher progesterone levels during the luteal phase than women with high aggression/irritability and fatigue. Additionally, aggression/irritability and fatigue correlated negatively with maximal progesterone value during the luteal phase. Results demonstrated a negative effect of low progesterone levels on premenstrual mood symptoms, such as aggressive behavior and fatigue in healthy reproductive-age women.	Statistically significant
Lovick et al. [43]	2017	46	Case control study	Progesterone	Evaluated not only the absolute concentrations of progesterone but also the kinetics of the change in progesterone concentration in relation to the development of premenstrual symptoms during the last 10 days of the luteal phase	In participants who developed symptoms of premenstrual distress, the daily saliva progesterone concentration remained stable during most of the mid-late luteal phase, before declining sharply during the last 3 days prior to the onset of menstruation. In contrast, progesterone concentration in asymptomatic women underwent a gradual decline over the last 8 days prior to menstruation. Neither the maximum nor minimum concentrations of progesterone in the two groups were related to the appearance or severity of premenstrual symptoms.	Statistically significant
Rapkin et al. [45]	2011	12 women with PMDD and 12 healthy women	Case control study	Blood samples were taken before each session for an assay of plasma estradiol and progesterone concentrations.	Positron emission tomography with [(18)F] fluorodeoxyglucose and self-report questionnaires to assess cerebral glucose metabolism. The primary biological end point was incorporated into regional cerebral radioactivity (scaled to the global mean) as an index of glucose metabolism. Relationships between regional brain activity and mood ratings were assessed.	There were no group differences in hormone levels in either the follicular or late luteal phase, but the groups differed in the effect of menstrual phase on cerebellar activity.	Women with PMDD but not comparison subjects showed an increase in cerebellar activity (particularly in the right cerebellar vermis) from the follicular phase to the late luteal phase (*p* = 0.003). In the PMDD group, this increase in cerebellar activity was correlated with worsening of mood (*p* = 0.018).
Redei et al. [49]	1995	10 women with confirmed PMS and 8 asymptomatic women	Case control study	Plasma levels of estradiol and progesterone were measured daily	Assessment of plasma ACTH levels in women with premenstrual syndrome (PMS) compared with asymptomatic controls.	Both estradiol and progesterone levels were consistently, but not significantly, higher throughout the cycle in PMS subjects compared with controls. From the follicular to the early luteal phase, estradiol levels were significantly higher in a previously defined PMS subgroup 2 with more severe symptoms throughout the cycle compared with both the less severe PMS subgroup 1 and controls. Progesterone levels were significantly and positively correlated with PMS symptoms along the entire menstrual cycle, preceding the symptoms by 5–7 days. These preliminary results provide support for the hypothesis that the presence of progesterone at early luteal phase levels is required for PMS symptoms to occur.	Statistically significant
Kaltsouni. et al. [63]	2021	35 women with PMDD	Double-blind, randomized, placebo-controlled	Determine the levels of estradiol, progesterone, testosterone, and cortisol.	Investigate the neural correlates of reactive aggression during the premenstrual phase in women with PMDD, randomized to a selective progesterone receptor modulator (SPRM) or placebo.	The findings contribute to defining the role of progesterone in PMDD symptomatology, suggesting a beneficial effect of progesterone receptor antagonism, and consequent anovulation, on top- down emotion regulation, i.e., greater fronto-cingulate activity in response to provocation stimuli.	Statistically significant
Bixo et al. [64]	2017	26 healthy women in a pharmacokinetic phase I study, and 126 women with PMDD in a phase II study.	Explorative randomized, double-blind, placebo-controlled study.	Subjects were randomized to treatment with UC1010 (10 or 16 mg) subcutaneously every second day during the luteal phase or placebo during one menstrual cycle.	Test whether inhibition of allopregnanolone by treatment with the GABAA modulating steroid antagonist (GAMSA) Sepranolone (UC1010) during the premenstrual phase could reduce symptoms of the premenstrual dysphoric disorder (PMDD).	This explorative study indicates promising results for UC1010 as a potential treatment for PMDD. The effect size was comparable to that of SSRIs and drospirenone-containing oral contraceptives. UC1010 was well tolerated and deemed safe.	Statistically significant
Bäckström et al. [65]	2021	206	A randomized, double-blind study	Treat PMDD patients with the GABAA receptor modulating steroid antagonist, sepranolone (isoallopregnanolone). Patients were administered sepranolone subcutaneously every 48 h during the 14 premenstrual days of three consecutive menstrual cycles.	Test the hypothesis that sepranolone is more effective than a placebo in reducing PMDD symptoms, presumably through sepranolone-induced inhibition or blockade of allopregnanolone action at the GABAA receptor in women with PMDD (Bäckström et al., 2011).	The results indicate that there is an attenuating effect by sepranolone on the symptoms, impairment, and distress in women with PMDD, especially at the 10 mg dosage. Sepranolone was well tolerated, and no safety concerns were identified.	Statistically significant
Bloch et al. [60]	2000	Eight women with and eight without a history of postpartum depression	Cross-sectional study	Estradiol and progesterone	Investigated the possible role of changes in gonadal steroid levels in postpartum depression by simulating two hormonal conditions related to pregnancy and parturition in euthymic women with and without a history of postpartum depression.	The data provide direct evidence in support of the involvement of the reproductive hormones, estrogen and progesterone, in the development of postpartum depression in a subgroup of women. Further, they suggest that women with a history of postpartum depression are differentially sensitive to the mood-destabilizing effects of gonadal steroids.	Statistically significant

## Data Availability

Not applicable.
